# Preferential formation of human heteromeric SK2:SK3 channels limits homomeric SK channel assembly and function

**DOI:** 10.1016/j.jbc.2022.102783

**Published:** 2022-12-09

**Authors:** Andrew S. Butler, Jules C. Hancox, Neil V. Marrion

**Affiliations:** School of Physiology, Pharmacology and Neuroscience, Medical Sciences Building, University of Bristol, University Walk, Bristol, United Kingdom

**Keywords:** electrophysiology, heteromer formation, KCNN2, KCNN3, membrane trafficking, pharmacology, potassium channels, preferential assembly, SK channels, stoichiometry, CaMBD, Calmodulin binding domain, HEK293, Human embryonic kidney, SK channels, Small conductance, calcium-activated potassium channels, UCL1684, 6,12,19,20,25,26-Hexahydro-5,27:13,18:21,24-trietheno-11,7-metheno-7H-dibenzo [b,n] [1,5,12,16]tetraazacyclotricosine-5,13-diium dibromide

## Abstract

Three isoforms of small conductance, calcium-activated potassium (SK) channel subunits have been identified (SK1-3) that exhibit a broad and overlapping tissue distribution. SK channels have been implicated in several disease states including hypertension and atrial fibrillation, but therapeutic targeting of SK channels is hampered by a lack of subtype-selective inhibitors. This is further complicated by studies showing that SK1 and SK2 preferentially form heteromeric channels during co-expression, likely limiting the function of homomeric channels *in vivo*. Here, we utilized a simplified expression system to investigate functional current produced when human (h) SK2 and hSK3 subunits are co-expressed. When expressed alone, hSK3 subunits were more clearly expressed on the cell surface than hSK2 subunits. hSK3 surface expression was reduced by co-transfection with hSK2. Whole-cell recording showed homomeric hSK3 currents were larger than homomeric hSK2 currents or heteromeric hSK2:hSK3 currents. The smaller amplitude of hSK2:hSK3-mediated current when compared with homomeric hSK3-mediated current suggests hSK2 subunits regulate surface expression of heteromers. Co-expression of hSK2 and hSK3 subunits produced a current that arose from a single population of heteromeric channels as exhibited by an intermediate sensitivity to the inhibitors apamin and UCL1684. Co-expression of the apamin-sensitive hSK2 subunit and a mutant, apamin-insensitive hSK3 subunit [hSK3(H485N)], produced an apamin-sensitive current. Concentration-inhibition relationships were best fit by a monophasic Hill equation, confirming preferential formation of heteromers. These data show that co-expressed hSK2 and hSK3 preferentially form heteromeric channels and suggest that the hSK2 subunit acts as a chaperone, limiting membrane expression of hSK2:hSK3 heteromeric channels.

Three different calcium-activated potassium channels (BK, IKCa, and SK) have been identified and characterized by their conductance, voltage- and calcium-sensitivity (1). The cloning of SK channel subunits showed that three isoforms are expressed in mammals (SK1-3; encoded by *KCNN1-3*) with different but overlapping tissue expression. For example, all three isoforms are present in pancreatic islet cells, whereas levels of SK1 transcript are negligible in atrial and endothelial cells where SK2 and SK3 mRNA is detected at a significantly higher level (2, 3, 4, 5). Within the brain, SK1 and SK2 protein and mRNA show considerable overlap in regions including the neocortex and hippocampus, whereas SK3 more prominently expresses in separate brain regions such as the basal ganglia and thalamus (6, 7). As a result of this broad tissue distribution, SK channels have been shown to play diverse roles including involvement in glucose secretion, neuronal excitability, vascular tone, memory and learning, and cardiac repolarization, particularly in the atria and failing heart (4, 8, 9, 10, 11). Consequently, SK channel modulation has been proposed as a novel mechanism for treatment of ataxia, Alzheimer’s and Parkinson’s disease, hypertension, and atrial fibrillation, amongst other disorders (4, 10, 12, 13, 14, 15, 16). There is therefore a clear need to understand how the different isoforms are expressed and can be inhibited.

The lack of SK channel isoform-selective inhibitors has hindered understanding of the function of these channels. The archetypal inhibitor, apamin, shows reasonable selectivity between current mediated by SK2 (IC_50_ of 30–140 pM) and the two other SK isoforms (0.6–8.0 nM for SK1 & 0.7–6.1 nM for SK3 (17, 18, 19, 20)), but it is hard to take advantage of this experimentally when more than one subtype is present. UCL1684, which displaces apamin from its binding site, also inhibits currents mediated by all SK channel isoforms and is unable to fully separate subtypes pharmacologically. This organic molecule inhibits SK1- and SK2-mediated current with similar potency (∼750 pM & ∼350 pM respectively), although SK3-mediated current is less sensitive with an IC_50_ of 2.5 to 9.5 nM (10, 21, 22, 23, 24). More recently, novel compounds (NS8593, AP14145 and AP30663) which reduce activation by rightward shifting calcium sensitivity have been developed, and these show little to no subtype selectivity (3, 25, 26).

Functional SK channels are tetramers, and it has been suggested that they favor assembling as heteromeric channels (27, 28). The preferential formation of heteromers between SK1 and SK2 subunits has been shown (28). In addition, co-expression of wildtype (WT) SK2 with an SK1 or SK3 isoform which does not conduct current due to selectivity filter mutations (GYG to AAA) prevents functional channels from forming, suggesting preferential formation of heteromeric channels even with a disrupted inner pore structure (27). This has important implications for the use of pharmacology to elucidate function.

Both human and animal models show that SK subunit expression levels are higher in the atria than the ventricles, and subsequently SK current inhibitors prolong the atrial action potential significantly more than the ventricular action potential (3, 10, 29, 30). SK2 and SK3 are the predominant transcripts in the human atria, with SK1 mRNA being 10-fold lower than those of the other isoforms (3, 31). Building upon studies which show that heteromeric SK1:SK2 channels preferentially form during co-expression, it has previously been proposed that atrial myocytes display functional heteromeric SK2:SK3 channels (10). In support of this, it has been shown that SK2 and SK3 co-localize to within Z-lines in isolated atrial cardiomyocytes (10, 32). The present study therefore aimed to investigate the ability of human SK2 (hSK2) and human SK3 (hSK3) subunits to form heteromeric channels. We demonstrate that co-expression of hSK2 and hSK3 subunits in HEK293 cells preferentially formed heteromeric channels of a fixed stoichiometry. Co-expression of hSK2 and hSK3 subunits produced a current that displayed intermediate sensitivity to inhibition by apamin or UCL1684 when compared with inhibition of homomeric channel current. The lack of pharmacology indicating homomeric channels demonstrates the preferential formation of heteromeric channels. Expression of functional heteromeric hSK2:hSK3 channels was contingent upon the level of hSK2 subunits.

## Results

### Co-expression with SK2 reduces surface expression of SK3

**Figure 1:**
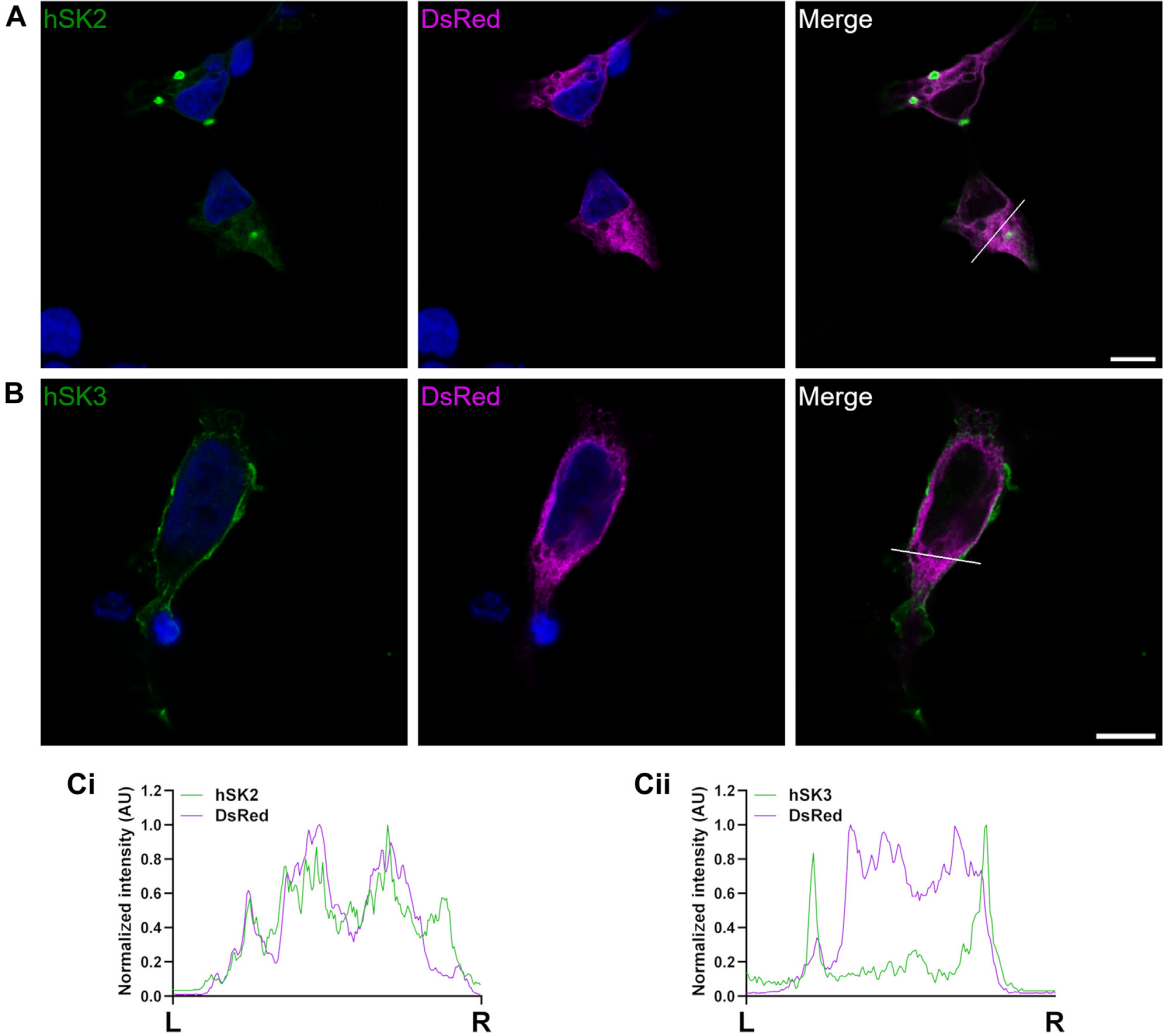
**Expression of DsRed with hSK2 or hSK3 subunits.***A* and *B,* representative images of cells co-expressing DsRed and hSK2 (*A*) or hSK3 (*B*) subunits. Fluorescence shows SK channels (*green*; *left*) or DsRed (*magenta*; centre) with DAPI (*blue*; *left* and *centre*). The *right-hand* images show a composite of the *green* and *magenta* channels only. Scale bars represent 10 μm. *White lines* overlaying the composite images highlight the cell section used for the intensity plots in C. *C,* intensity plots showing the relative distribution of DsRed with hSK2 (i) and hSK3 (ii) subunits. Plots were calculated along the lines shown from *left* (L) to *right* (R). SK channels, small-conductance, calcium-activated potassium channels.

Immunostaining for expressed hSK2 subunits revealed a diffuse pattern of staining which showed a strong degree of correlation between hSK2 and the ER marker DsRed2ER (DsRed) (Fig. 1*A*). In contrast, cells transfected with hSK3 subunits exhibited a distinctive expression pattern at the cell membrane (Fig. 1*B*). This difference in localization was confirmed by plotting the signal intensity along a line, across a section of cells. As illustrated in Fig. 1*C*, hSK3 subunits showed clear peaks at the cell edge. In contrast, expression of hSK2 subunits produced even distribution of subunits within the cell.

**Figure 2:**
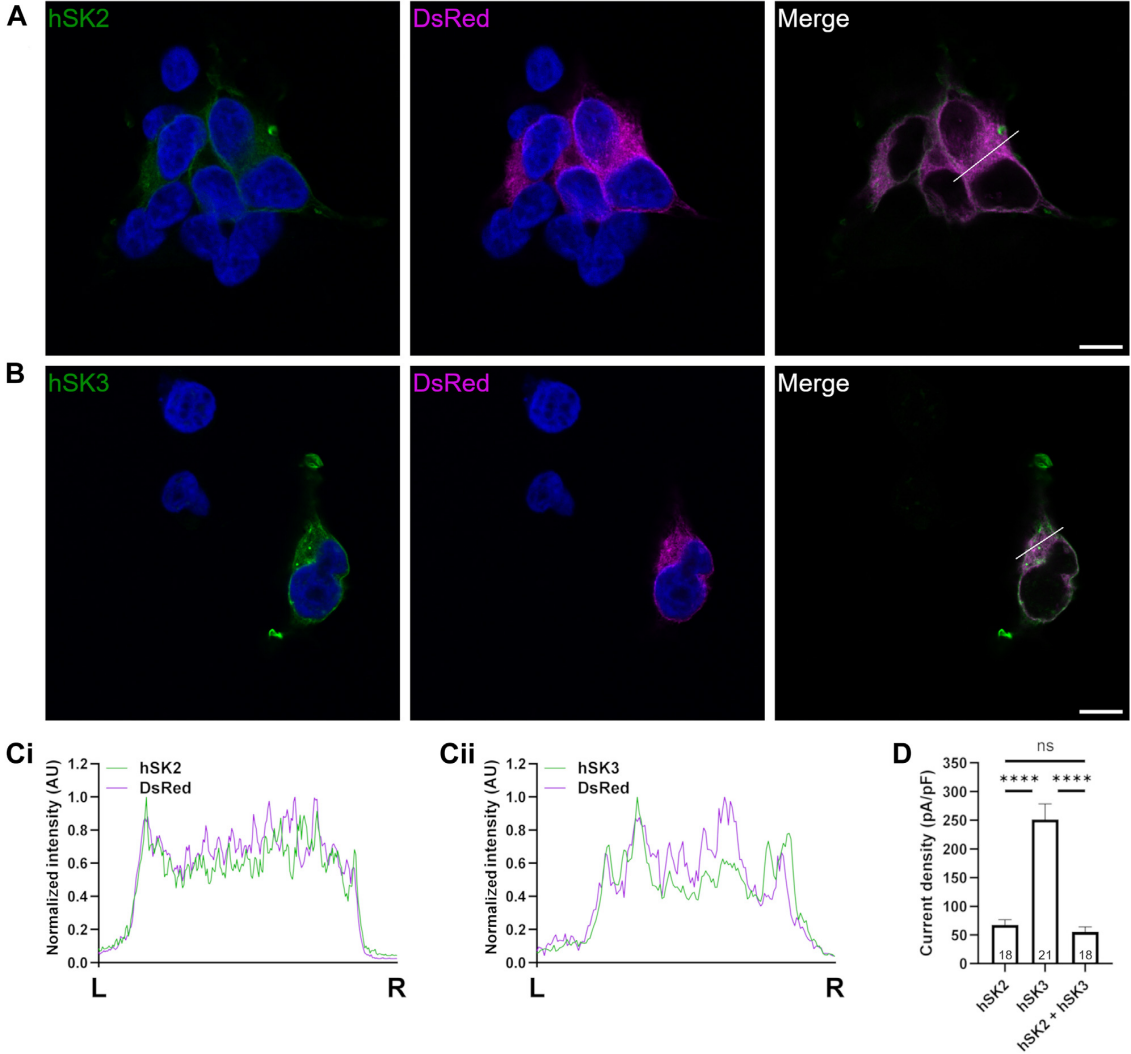
**Expression of DsRed with hSK2 and hSK3 subunits.** Representative images of cells co-expressing DsRed, hSK2, and hSK3 subunits. *A* and *B*, fluorescence shows hSK2 (*A*; *green*; left), hSK3 (*B; green*; *left*), or DsRed (*magenta*; *centre*) with DAPI (*blue*; *left* and centre). The right-hand images show a composite of the *green* and *magenta* channels only. Scale bars represent 10 μm. *White lines* overlaying the composite images highlight the cell section used for the intensity plots in C. *C,* intensity plots showing the relative distribution of DsRed with hSK2 (i) and hSK3 (ii) subunits. Plots were calculated along the lines shown from *left* (L) to *right* (R). *D,* homomeric hSK3 currents were larger than those of homomeric hSK2 or during co-expression of both isoforms. Currents were elicited during whole-cell voltage clamp using voltage ramps and measured at −20 mV. Data are presented as mean ± SD. SK channels, small-conductance, calcium-activated potassium channels.

Immunolabeling of hSK2 subunits in cells co-expressing hSK2 and hSK3 protein showed a similar pattern of expression as observed during expression of hSK2 alone. No distinctive membrane expression was present, and a relatively consistent level of expression was exhibited across the cells (Fig. 2*A*). As seen when hSK2 subunits were expressed alone, signal intensity plots showed a strong correlation between the ER and hSK2 (Fig. 2*C*i). In contrast to the clear membrane labeling observed for hSK3 subunits when expressed alone in HEK293 cells, hSK3 subunits labeling showed that the intracellular distribution during co-expression with hSK2 subunits. Co-expression with hSK2 subunits dictated that hSK3 subunit distribution was similar to that of hSK2 subunits, with labeling showing a reduction in membrane staining and more diffuse distribution (Fig. 2, *B* and *C*ii).

These data suggested that co-expression with hSK2 subunits reduced hSK3 protein reaching the cell membrane, with a higher proportion residing within the ER. Whole-cell patch clamp experiments were performed to investigate whether differences in intracellular distribution of SK channel subunits affected the magnitude of functional current. Whole-cell hSK2 currents were significantly smaller than those carried by hSK3 (Fig. 2*D*; 67.4 ± 9.2 pA/pF *versus* 251.4 ± 27.0 pA/pF; *n* = 18 & 21; *p* < 0.0001). Co-expression of the two isoforms resulted in a current which had an amplitude similar to that seen with hSK2 subunit expression alone (59.8 ± 9.4 pA/pF; *n* = 24; *p* > 0.05) and was significantly smaller than the current amplitude observed when homomeric hSK3 channels were expressed (*p* < 0.0001). These observations are consistent with the changes in cellular distribution shown in Figures 1 and 2, suggesting that expression on the cell surface and therefore current density is regulated by expression levels of hSK2 subunits.

### SK2 and SK3 form heteromeric channels

**Figure 3:**
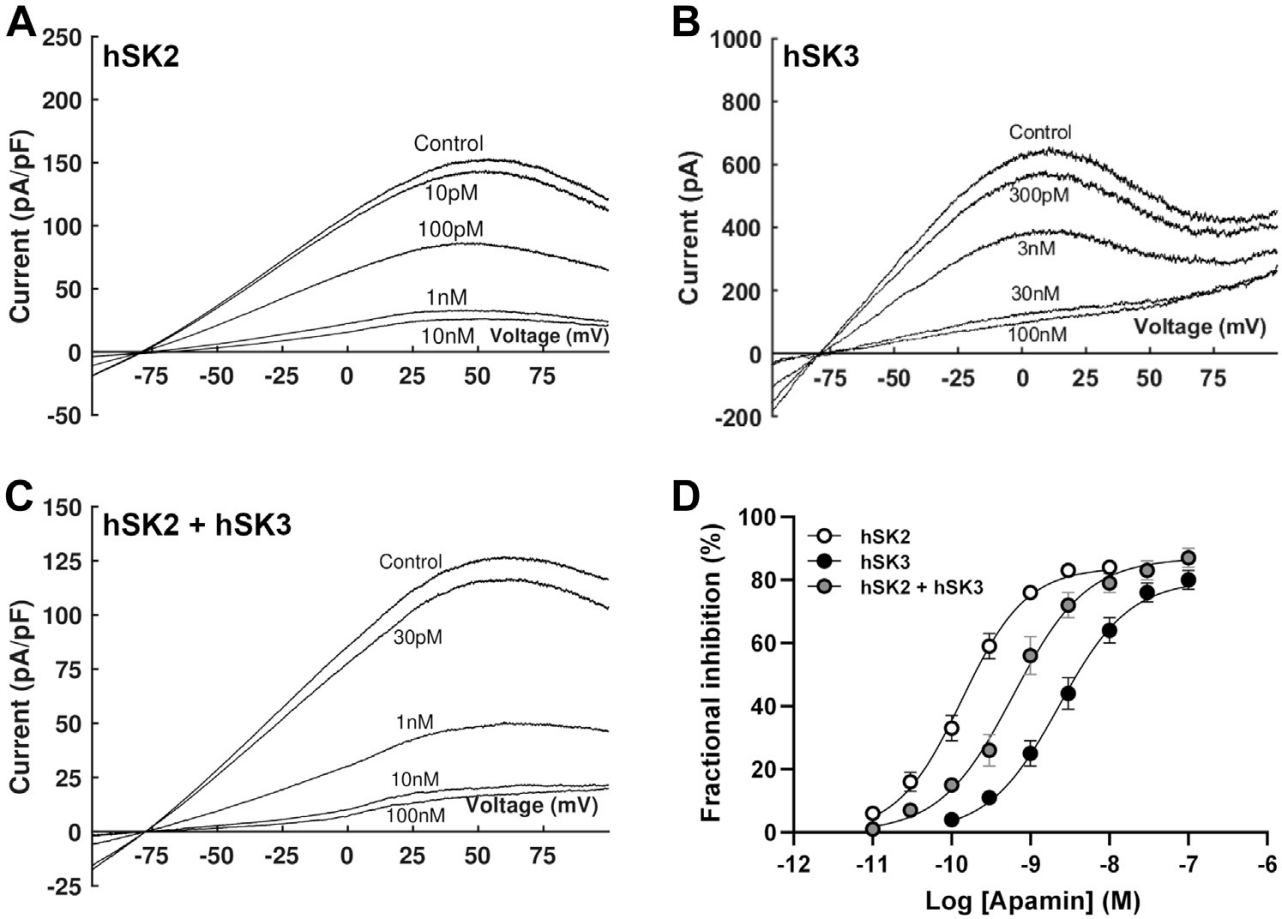
**Sensitivity of WT SK channels to apamin.***A–C*, representative traces following expression of hSK2 (*A*; *n* = 7), hSK3 (*B*; *n* = 7) or hSK2 + hSK3 (*C*; *n* = 7) subunits evoked by a voltage ramp from −100 mV to +100 mV (1 s duration). Under all conditions, current was inhibited by incrementally increasing concentrations of apamin. *D,* concentration–inhibition relationships show hSK2-mediated current to be more sensitive to apamin than hSK3-mediated current. Co-expression of both isoforms produced current with an intermediate sensitivity. SK channels, small-conductance, calcium-activated potassium channels.

Expression of homomeric hSK2 or hSK3 subunits produced currents that reversed at -75.9 ± 0.8 mV and -77.9 ± 1.8 mV, respectively (*p* > 0.05; *n* = 13 for each), with a current-voltage relationship exhibiting negative slope at positive voltages (Fig. 3, *A* and *B*). Application of increasing concentrations of apamin led to hSK2 inhibition with an IC_50_ of 141 ± 21 pM (*n* = 7; Fig. 3, *A* and *D*). Expressed hSK3—mediated current was significantly less sensitive to apamin and was inhibited with an IC_50_ of 2.62 ± 0.36 nM (Fig. 3, *B* and *D*; *n* = 7; *p* < 0.0001). The Hill slope for inhibition by apamin in each channel was similar (1.12 ± 0.09 *versus* 1.08 ± 0.06, *p* > 0.05). Co-expression of hSK2 and hSK3 subunits produced an inwardly rectifying current which reversed at -78.6 ± 0.9 mV (*n* = 14; *p* > 0.05 *versus* hSK2 & hSK3; Fig. 3*C*). Construction of a concentration-inhibition relationships showed current to be inhibited by apamin with an IC_50_ intermediate to those of the two homomeric channel currents (650 ± 126 pM; *n* = 7), which was best fit by a monophasic Hill equation (Hill slope 1.18 ± 0.19; *p* > 0.05 *versus* homomers; Fig. 3*D*).

**Figure 4:**
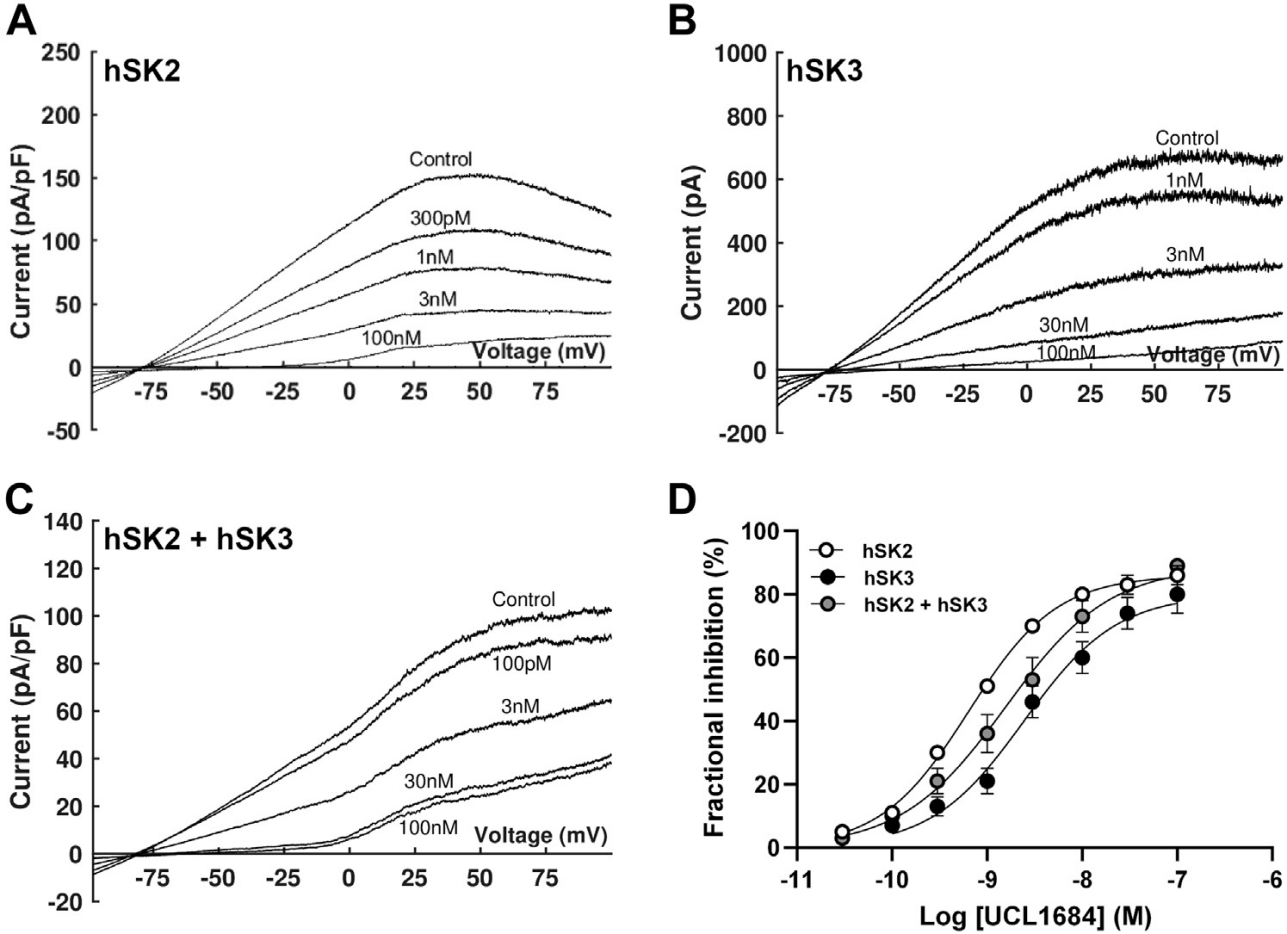
**Sensitivity of WT SK channels to UCL1684.***A–C,* representative traces following expression of hSK2 (*A*; *n* = 6), hSK3 (*B*; *n* = 7), or hSK2 + hSK3 (*C*; *n* = 9) subunits evoked by a voltage ramp from −100 mV to +100 mV (1 s duration). Under all conditions, current was inhibited by incrementally increasing concentrations of UCL1684. *D,* concentration–inhibition relationships show hSK2-mediated current to be more sensitive to UCL1684 than hSK3-mediated current. Co-expression of both isoforms produced current with an intermediate sensitivity. SK channels, small-conductance, calcium-activated potassium channels.

Expressed homomeric hSK2-mediated current was more sensitive to inhibition by UCL1684 than current mediated by hSK3 channels (Fig. 4; IC_50_: 595 ± 79 pM *versus* 2.72 ± 0.7 nM; n = 6 & 7; *p* < 0.05; Hill slope 1.1 ± 0.05 *versus* 1.06 ± 0.11, *p* > 0.05). Co-expression of hSK2 and hSK3 subunits produced a current with an intermediate sensitivity to UCL1684 (2.37 ± 0.63 nM), the concentration–response relation for which was best fit by a monophasic curve and showed no difference in Hill slope compared with homomeric channels (0.97 ± 0.04; *p* > 0.05; Fig. 4, *C* and *D*). These data indicate that co-expression of hSK2 and hSK3 channel subunits preferentially forms heteromeric channels that display an intermediate sensitivity to inhibitors.

### Mutant hSK3(H485N) subunits express in the same manner as WT hSK3 subunits

**Figure 5:**
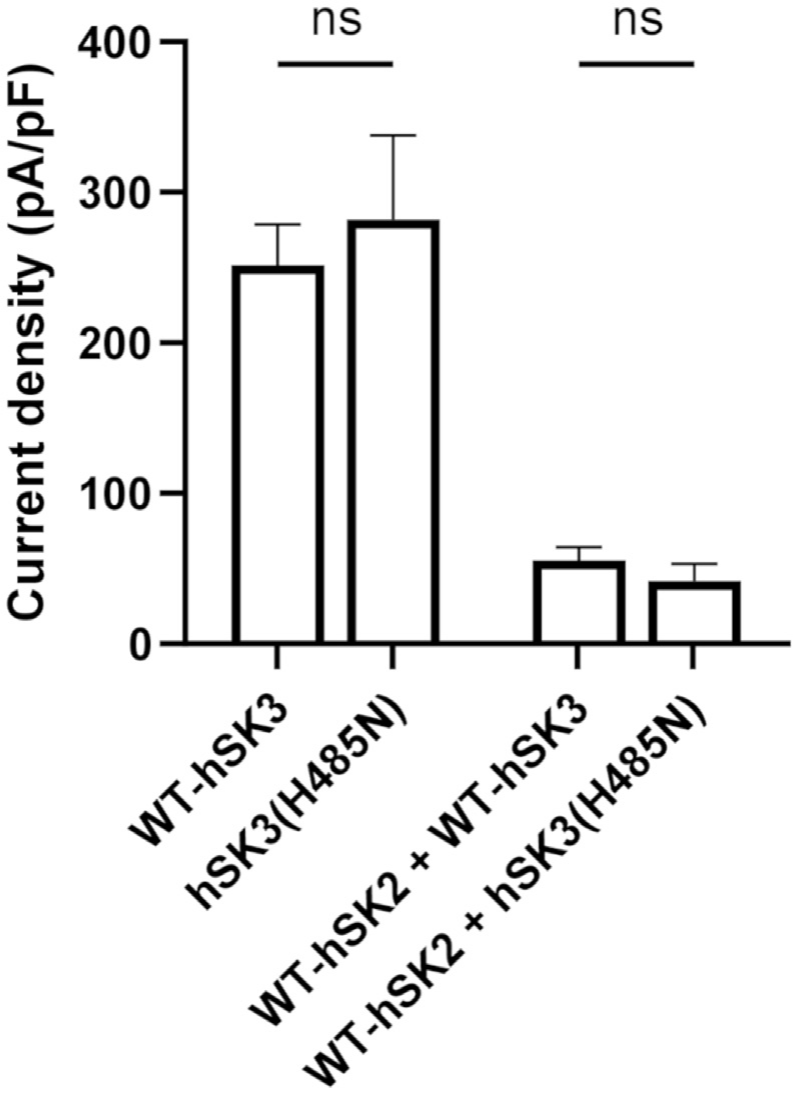
**Whole-cell current amplitudes of channels containing hSK3(H485N) subunits**. The outer pore histidine-to-asparagine mutation in hSK3 (H485N) had no effect on current size when expressed as a homomer or with WT-hSK2. Currents were elicited during whole-cell voltage clamp using voltage ramps and measured at −20 mV. Data are presented as mean ± SD.

Previous experimental work has shown that channel inhibition by apamin occurs when the toxin binds simultaneously to the S3-S4 extracellular loop (SYA/SYT motif in hSK2/hSK3) of one subunit and an outer pore histidine (H336/H485) of an adjacent subunit (21, 28). UCL1684 displaces apamin from its binding to the channel outer pore (21, 33). Mutation of the outer pore histidine residue produces a subunit that is insensitive to inhibition by apamin or UCL1684 when expressed as a homomeric channel (19). Heteromeric channel formation was studied by co-expression of WT and outer pore mutant subunits. Expression of hSK3(H485N) subunits produced whole-cell currents of a similar amplitude to that seen when WT-hSK3 subunits were expressed alone (281.7 ± 56.0 pA/pF; *n* = 16; *p* > 0.05). These were reduced in amplitude by co-expression with WT-hSK2 subunits, and the amplitude was not significantly different from that observed with expression of WT channels (41.9 ± 11.7 pA/pF; *n* = 19; *p* > 0.05; *n* = 16 Fig. 5).

### SK2:SK3 heteromers form with a fixed stoichiometry

**Figure 6:**
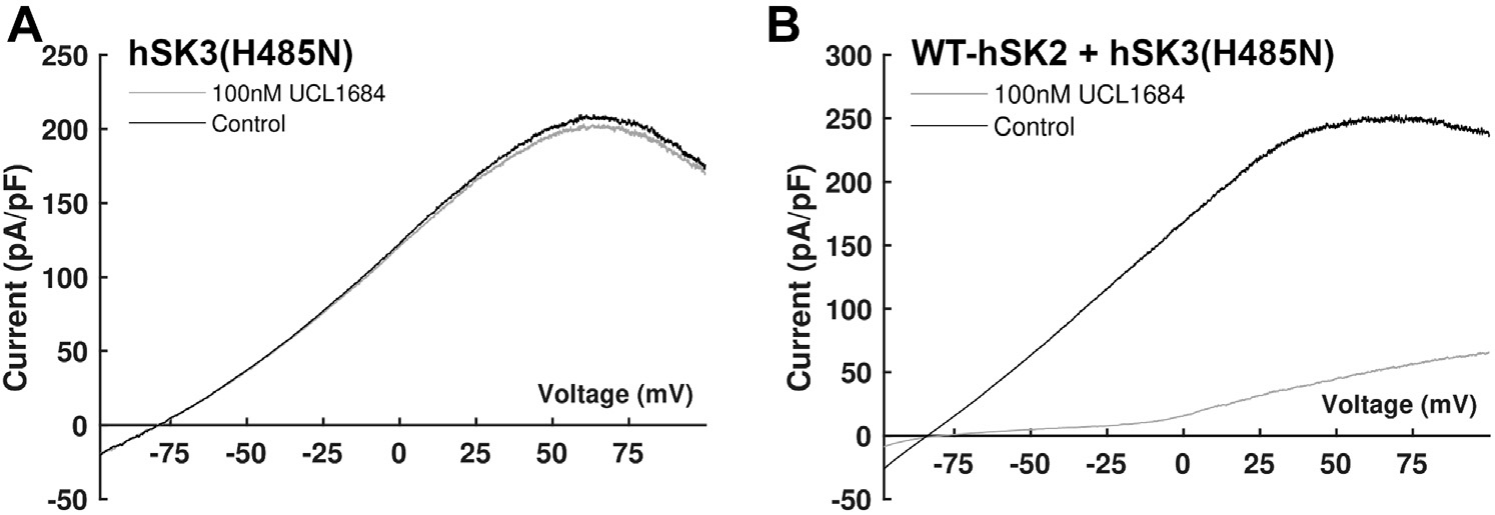
**UCL1684 sensitivity of cells expressing hSK3-H485N subunits alone or with WT-hSK2 subunits.***A* and *B,* 100 nM UCL1684 did not inhibit hSK3(H485N)-mediated current (*A*) but maximally inhibited current during co-expression of hSK3(H485N) and WT-hSK2 (*B*; 80 ± 4%; *n* = 8). Control traces are shown in *black* and traces after application of 100 nM UCL1684 are in *gray*.

**Figure 7:**
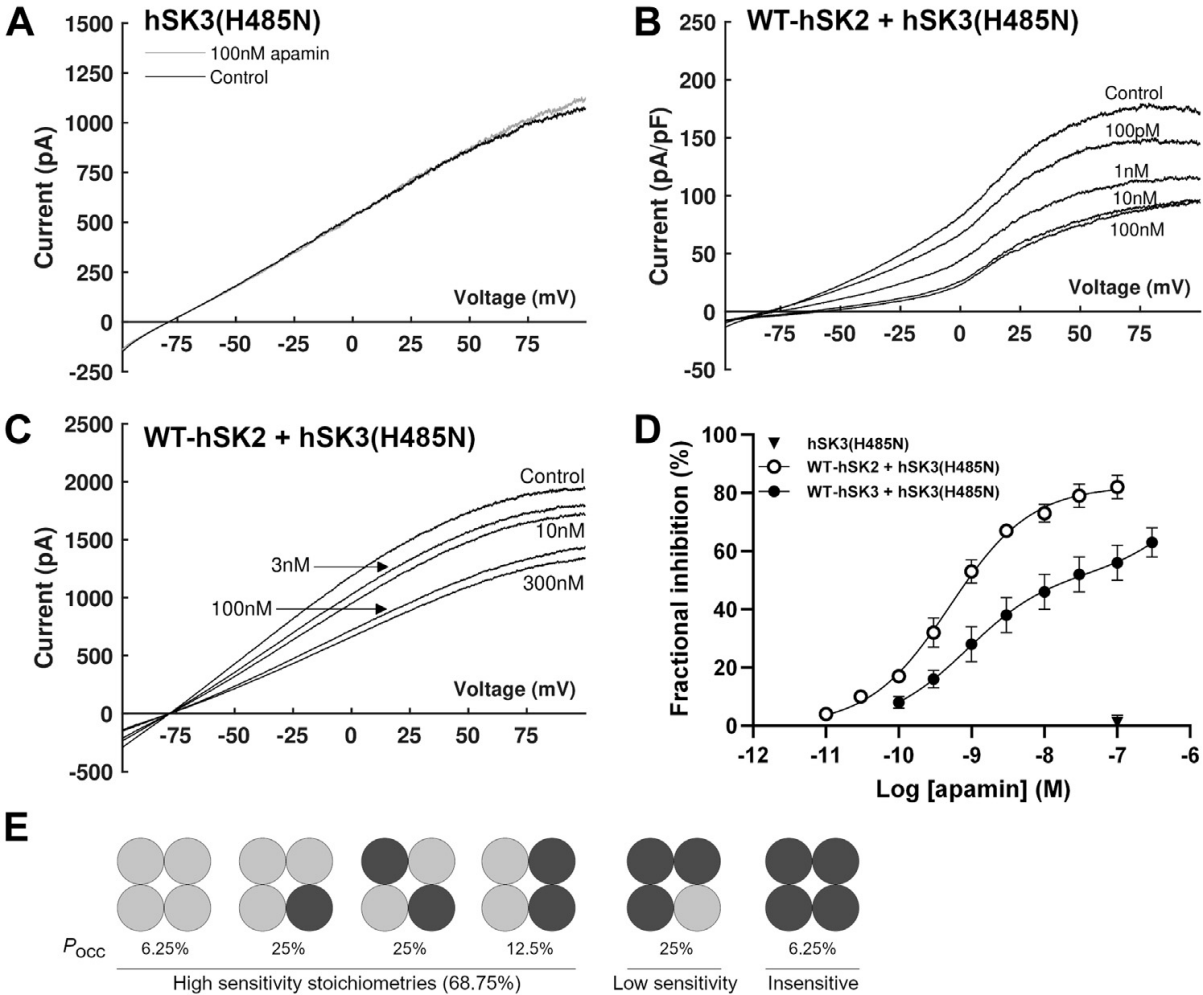
**Apamin sensitivity of cells expressing hSK3(H485N) subunits alone or with WT subunits.***A–C*, representative recordings following application of apamin to cells expressing hSK3(H485N) only (*A*; *n* = 5); WT-hSK2 and hSK3(H485N) (*B*; *n* = 7), or WT-hSK3 and hSK3(H485N) (*C*; *n* = 7). *D,* concentration–inhibition relationship for apamin during each expression condition. Expression of hSK3(H485N) alone (▼) produced a current which was insensitive to apamin. Co-expression with WT-hSK2 (○) produced a response which was best fit by a monophasic Hill equation, whilst co-expression with WT-hSK3 (●) produced a response which was best fit by a biphasic Hill equation. *E,* the probability of occurrence (*P*_occ_) for each possible channel stoichiometry, calculated assuming the likelihood of inclusion of WT-hSK3 (*light gray*) or hSK3(H485N) (*dark gray*) subunits was equivalent.

In agreement with previous work (19, 21), channels produced from expression of hSK3(H485N) subunits alone were insensitive to inhibition by either UCL1684 or apamin (Figs. 6*A* and 7*A*). Co-expression of WT-hSK2 and hSK3(H485N) subunits produced a current which was maximally inhibited by 100 nM UCL1684 to the same degree as observed with WT heteromeric channel current (80 ± 4% *versus* 88 ± 2%; see Fig. 6*B* *versus* Fig. 4*D* respectively; *n* = 6 & 8; *p* > 0.05). Similarly, fractional inhibition by 100 nM apamin was not significantly reduced when hSK3(H485N) subunits were co-expressed with WT-hSK2 subunits compared with when WT-hSK3 subunits were used for co-transfection (82 ± 4% for H485N *versus* 87 ± 3% for WT; see Figs. 7, *B* and *D* and 3*D*, respectively; *n* = 7 for both; *p* > 0.05), demonstrating that no homomeric hSK3(H485N) channels were present during co-expression with hSK2 subunits. This co-expression of WT-hSK2 and hSK3(H485N) subunits produced a channel current which was inhibited by apamin with an IC_50_ of 588 ± 183 pM (*p* > 0.05 *versus* co-expression of WT channels; Fig. 7, *B* and *D*). This was best fit by a monophasic relation, indicating a single population of channels and the preferred formation of heteromeric hSK2:hSK3(H485N) channels.

In contrast, co-expression of WT-hSK3 with hSK3(H485N) subunits produced concentration-inhibition responses to apamin which were best fit by a biphasic curve, with a high sensitivity component matching the IC_50_ of WT-hSK3 channel current (1.60 ± 0.92 nM; *p* > 0.05) and a low sensitivity component with an IC_50_ of 25.5 ± 11.4 nM (*n* = 7; Fig. 7, *C* and *D*). Concentrations of up to 300 nM failed to fully inhibit current, suggesting that a population of homomeric hSK3(H485N) channels were also present (19).

Binomial distribution predicts that 69% of channels would be inhibited with a high sensitivity if the probability of incorporating WT-hSK3 or hSK3(H485N) subunits is equivalent (Fig. 7*E*). This occurs as each molecule binds to the outer pore of one subunit and the S3-4 loop in an adjacent subunit. A low sensitivity component (25%) and an apamin-insensitive component (6%) are also predicted (Fig. 7*E*). The high sensitivity component of the biphasic curve in Fig. 7*D* constituted ∼65% of total inhibition during co-expression of WT-hSK3 and hSK3(H485N) subunits and therefore likely represents channels with at least two apamin-sensitive subunits. Low sensitivity inhibition accounted for ∼26% of current and represents channels formed with three hSK3(H485N) subunits. These data showed that co-expression of WT and mutant hSK3 subunits produced a mixed population of channels, with each channel containing none to four mutant subunits. In contrast, co-expression of WT-hSK2 and hSK3(H485N) subunits produced only one population of channels which exhibit a fixed subunit stoichiometry.

## Discussion

### SK2 and SK3 form heteromers with a fixed stoichiometry

The three cloned SK channel subunits (SK1-3) are ∼90% homologous between the beginning of S1 and the distal end of the calmodulin-binding domain (CaMBD) in the C terminus, with the residues outside of this region showing little to no homology (34). Heteromer formation in SK channels relies on C-terminal interactions distal to the CaMBD and is species specific (27, 28). Co-expression of rSK1 and rSK2, or hSK1 and hSK2 subunits, leads to the preferential species-specific assembly of heteromeric channels (28). Apamin concentration-inhibition relationships for SK1:SK2 heteromers were best fit by a monophasic Hill distribution and showed a sensitivity intermediate to those observed with homomeric channels (28). These properties demonstrate the preferential formation of heteromeric SK1:SK2 channels. The same preferential formation of heteromeric channels was observed in the present study upon co-expression of hSK2 and hSK3 channel subunits.

Heteromeric channel conformation was confirmed by co-expressing hSK3(H485N) with WT-hSK3 or WT-hSK2 channel subunits. As nonconserved C terminus sequences are key in channel assembly, when apamin-sensitive and apamin-insensitive subunits of the *same* isoform are co-expressed (*e.g.*, hSK2 & hSK2(H337N); rSK2 & rSK2(YA246/7LV); hSK3 & hSK3-ex4), subunits form channels with stoichiometries which approximately follow the binomial distribution (10, 19, 21). This means that each channel contains a variable number of apamin-insensitive subunits and consequently concentration–inhibition relationships for apamin are bi-phasic, demonstrating a high sensitivity component (<3 nM), a very low sensitivity component (20–50 nM) and a reduction maximal inhibition (19, 21). Concentration-inhibition relationships for current recorded from cells co-expressing WT-hSK3 and hSK3(H485N) subunits exhibited this bi-phasic inhibition curve alongside a population of apamin-insensitive channels. (Fig. 7*D*). The proportion of current inhibited with low (25 nM) and high (1.6 nM) sensitivities demonstrated that channels formed with a subunit organization which followed a binomial distribution. In contrast, co-expression of WT-hSK2 and hSK3(H485N) subunits produced a current which was inhibited by apamin with a high sensitivity and best fit by a monophasic curve (Fig. 7, *B* and *D*). This is consistent with previously published data showing that co-expression of the apamin-insensitive rSK1 with rSK2 subunits produced a single population of channels which were inhibited by apamin with a high sensitivity (28).

During co-expression of hSK2 and either WT-hSK3 or hSK3(H485N), no evidence of homomeric channels was present, suggesting mandatory formation of heteromeric channels. Taken together, these data show that hSK2 and hSK3 co-assemble to form channels of a fixed stoichiometry. The formation of heteromeric SK2:SK3 channels has been suggested to occur previously in both native tissue (10, 32) and in heterologous expression systems (10, 35). The present data extend previous work by demonstrating that this formation is *preferential*, produce channels with a fixed stoichiometry, and also lead to an altered pattern of subcellular distribution. Co-expression of WT-hSK2 with hSK3(H485N) subunits showed a nonsignificant trend toward reducing co-operativity of inhibition (0.94 ± 0.14 *versus* 1.18 ± 0.19). The cooperative inhibition by apamin requires *adjacent* apamin-sensitive subunits to be present (19, 21, 28), and so, these data suggest that heteromers likely form with *non-adjacent* SK2 subunits. Taken together, these data show that when subunits of the same isoform are co-expressed, they assemble randomly into functional channels. Co-expression of different isoforms, however, results in preferential assembly of heteromeric hSK2:hSK3 channels with a fixed stoichiometry and likely containing nonadjacent SK2 subunits.

### Expression of heteromeric hSK2:hSK3 channels

Immunocytochemistry revealed prominent SK channel expression at the membrane when hSK3 subunits were expressed alone. In contrast, when hSK3 subunits were co-expressed with hSK2 subunits, the intracellular distribution was diffused and mirrored by the intracellular distribution of expressed hSK2 subunits (Figs. 1 and 2). As homomeric SK2 and SK3 channels exhibit the same single channel conductance and calcium sensitivity, a reduction in the number of channels trafficked to the cell membrane would be predicted to lead to a reduced current amplitude (8, 36, 37, 38, 39). In agreement with this, the amplitude of co-expressed heteromeric hSK2:hSK3 channel current was similar to that observed when homomeric hSK2 subunits were expressed and significantly smaller than that seen during expression of homomeric hSK3 subunits (Fig. 2*D*). It is suggested that the hSK2 subunit appears to act as a chaperone and controls the surface expression of hSK3 subunits during co-expression. These data are consistent with previous studies showing that the rSK2 subunit acts as a molecular chaperone for rSK1 subunits and augments its subcellular localization (40, 41).

In comparison with homomeric hSK3 channels, the reduced proportion of hSK2 homomers and hSK2:hSK3 heteromers which reach the membrane may stem from an ER-retention sequence (^563^RRRR) which is present on C terminus of the SK2 subunit but absent in the SK3 subunit (42, 43). A second ER-retention sequence (^490^YxxΦ; where ‘x’ represents any amino acid and Φ is an amino acid with a hydrophobic side chain) is present in both subunits but lies within the predicted coiled-coil region critical for channel assembly. This could therefore be masked during tetramerization or may contribute to the proportion of hSK3 channel homomers which do localize to the ER (27, 42). Localization to the ER leads to different functional role of these channels. Recent work in cultured endothelial cells has shown that surface expression of SK2 subunits was comparatively reduced, and they were expressed more highly in the mitochondria and ER than either SK1 or SK3 subunits (36). Due to this sublocalization, SK2 plays a role in protection against cell-death triggered by oxidative and ER stress (36). Similarly, the mitochondrial inner membrane in neuronal and cardiac cells contains SK2 and SK3 subunits which protect against damage trigged by ischemia and reperfusion (44, 45, 46). In addition, exposure to hypoxia and reoxygenation increases neuronal expression of SK2 and SK3 subunits, but not SK1 subunits (47). Our data suggest that these protective effects may benefit from SK2-mediated trafficking of heteromeric channels. Heteromer formation, and the subsequent trafficking to sites other than the cell membrane, can also be predicted to regulate the contribution of SK-mediated current to the atrial action potential. Dysregulation of the ratio between SK channel isoforms, as has been reported in AF, may lead to unexpected consequences for repolarizing currents due to a redistribution of functional channels (3, 48, 49).

Analysis of human atrial tissue has shown low levels of SK1 subunit mRNA and much higher levels of SK2 and SK3 subunit mRNA in tissues from patients both with and without atrial fibrillation (3). Confocal immunohistochemistry work from our laboratory showed significant co-localization of SK2 and SK3 protein at the periphery of atrial myocytes (10, 32). The findings of the present study make it likely that such co-localization represents the presence of heteromeric SK2:SK3 channels (10, 32). In atrial myocytes and in heterologous expression systems, membrane expression of SK2 is increased by interactions with structural proteins, including junctophilin 2 (JPH2), α-actinin2 (ACT2), and filamin A (FLNA). ACT2 interacts with the CaMBD, which is highly conserved between SK isoforms, but JPH2 and FLNA interact with nonconserved regions of the N and C termini, respectively (50, 51, 52, 53). Consequently, effects of these trafficking proteins on the surface localization of SK channels may vary between SK channel isoforms. It can be expected that heteromer formation may therefore affect these interactions, suggesting that heteromerization could alter the subcellular distribution of channels.

Evidence suggests overall that preferential heteromerization of SK subunits produces channels with distinct cellular roles. Although this occurs during co-expression of SK2 and either SK1 or SK3 subunits, co-expression of SK1 with SK3 subunits does not lead to the preferential formation of functional heteromeric channels (our unpublished data + (35)). The SK2 subunit therefore appears to be critical in the formation of heteromeric channels and in chaperoning subunits of each isoform to specific subcellular locations. The conservation of an ER-retention sequence within the coiled-coil domain of SK1-3 subunits suggests that heteromerization occurs during initial protein processing in the ER/Golgi apparatus, thus restricting the role of homomeric channels. Although apamin and UCL1684 cannot distinguish between homomeric and heteromeric channels in a way which allows pharmacological targeting of SK2:SK3 channels, increased understanding of the way in which heteromeric channels are formed and localized within cells may be beneficial in the treatment of the disorders with which SK channels have been associated.

## Experimental procedures

### DNA constructs and cell culture

Human SK2 (NM_021614.2, OMIM 605879) in pcDNA3 and human SK3 (NM_002249.6, OMIM 602983) in pcDNA3.1-c-DYK were used for all experiments. hSK2 was a generous gift from Dr Palle Christophersen (NeuroSearch A/K, Denmark) and hSK3 was purchased from GenScript. The SK3(H485N) mutation was made using the QuikChange II XL Site-Directed Mutagenesis Kit (Agilent). Successful mutagenesis was confirmed by Sanger sequencing of the complete open reading frame (Eurofins Genomics). Enhanced green fluorescent protein (eGFP) in pEGFP-C3 and DsRed (pDsRed2-ER) were purchased from Clontech.

HEK293 cells were maintained at 37 °C and 5% CO_2_ in Dulbecco’s minimum essential medium supplemented with 10U/ml penicillin/streptomycin and 10% FBS (All from Gibco). Cells were replated into 35 mm tissue culture dishes and transfected using 3 μl Lipofectamine 2000 (Invitrogen) at least 24 h later and once they reached 80 to 90% confluency. For the study of homomeric currents, each dish was transfected using 1 μg ion channel, and 200 ng eGFP was included as a marker of successful transfection. Due to the difference in current size between expression of SK2 and SK3 subunits, only 100 ng SK3 was used for the study of heteromeric currents, and 400 ng empty vector (pcDNA3.1) was included in the transfection mixture to maintain a total of 1.2 μg DNA. The same quantities were used for cotransfection of WT-hSK2 and hSK3(H485N). These altered quantities of DNA did not affect current size (see Fig. S1) but produced consistent data. For co-transfection of WT-hSK3 and hSK3(H485N), 500 ng of DNA encoding each subunit was used. Cells were dissociated 16 to 24 h after transfection and plated onto glass coverslips. Electrophysiological recordings were made 40 to 48 h after transfection. Data were collected from a minimum of three different transfections.

### Immunocytochemistry

Transfections were performed as above but DsRed was co-expressed with the subunits of interest in place of eGFP in order to label the endoplasmic reticulum. Additionally, cells were plated onto poly-L-lysine (Sigma Aldrich)–coated glass. Cells were washed in ice-cold PBS supplemented with 1 mM MgCl_2_ and 0.1 mM CaCl_2_ (PBS^+^) and fixed in 4% PFA for 20 min before being permeabilized with Triton X-100 (3 × 5-min incubations; Santa Cruz). Cells were then blocked with 2.5% horse serum (Vector Laboratories) before a 1-hour incubation in primary antibody (Alomone APC-025 or -028) diluted 1:200 in blocking buffer. Following three washes in PBS^+^, secondary antibodies were then applied (Vector Laboratories, DK-8818) for 1 hour, before cells were incubated in 1.2 μM DAPI (Sigma) for 5 min after excess secondary antibody was washed off with PBS^+^. Coverslips were mounted onto slides using ProLong Gold Antifade reagent (Invitrogen), which was allowed to set at overnight before being stored at 4 °C. All steps were performed at room temperature.

Staining was imaged using a 63 × oil immersion lens with a Leica DM1600 confocal microscope. Fluorophores were excited at 488 (SK) or 568 nm (DsRed), and emission from a single confocal plane of 1 nm was detected. Images were processed and analyzed using FIJI (54). Signal intensity plots were calculated by selecting a representative region of interest across a cell which avoided the nucleus (shown as white lines on Figs. 1 and 2). Intensities were normalized to the maximum intensity measured across the section. Representative cells were selected for Figs. 1 and 2 following imaging of at least six areas of coverslips during each transfection condition, and transfections were repeated three times to ensure consistency.

### Electrophysiology

Glass coverslips were placed into a recording chamber and superfused at room temperature with a solution of composition (in mM): NaCl (138), KCl (4), Hepes (10), D-glucose (10), MgCl_2_ (1.2), CaCl_2_ (2.5) and titrated to pH 7.4 with NaOH. Glass patch pipettes (WPI, 1B150F-3) were pulled to a resistance of 2 to 3.5 MΩ and filled with intracellular solution containing (in mM): K-aspartate (97), KCl (20), Na_2_-ATP (1.5), Hepes-Na (10), EGTA (10), CaCl_2_, and MgCl_2_ and titrated to pH 7.2 with KOH. Homomeric currents were recorded in the presence of 500 nM free Ca^2+^ and 1 mM free Mg^2+^. Concentrations of Mg^2+^ and Ca^2+^ were calculated using Max Chelator V8 (55). Although homomeric currents were stable under these conditions during recordings, currents ran down considerably following co-expression of hSK2 and hSK3 subunits. Using the intracellular solution above but adjusted to contain 1 μM free Ca^2+^ and 50 μM free Mg^2+^ stabilized currents without affecting current size (data not shown).

Channel current was elicited using voltage ramps running from 100 mV to +100 mV (1 s duration). Where current amplitudes are reported (Figs. 2*D* and 5), these were measured using whole-cell voltage clamp and recorded at -20 mV during this voltage ramp. This voltage was selected in order to reduce interference from endogenous voltage-gated potassium channels and to ensure that drug-induced changes in rectification did not affect the analyzed current amplitude. Additionally, in some cells (typically those expressing hSK3) currents were too large to clamp at positive voltages so the use of −20 mV allowed for current density measurements to made in the whole-cell configuration. This was important for accurately comparing current amplitude with that mediated by hSK2 channels. When currents were too large to be clamped at positive voltages in the whole-cell configuration, outside-out patches were pulled instead for pharmacological experiments, and data from different patch configurations were pooled as it did not affect pharmacology.

Current was recorded using an Axopatch 200B amplifier (Axon instruments) and filtered at 1 kHz using a low pass Bessel filter (eight-pole; Frequency Devices Inc.) before sampling at 10 kHz using Pulse (HEKA). Electrode resistance was compensated for by at least 70% (typically 2–4 MΩ) when the whole-cell configuration was used. Liquid junction potentials were not corrected for.

### Pharmacology

Apamin and UCL1684 (both Bio-Techne) were diluted to 100 μM in ddH_2_O and DMSO, respectively, with aliquots stored at −20 °C. Aliquots were thawed on the day of use and diluted further in the external solution described above. Aliquots were sequentially added to the superfusate to obtain concentrations ranging from 10 pM to 300 nM. Concentration–inhibition relationships were fit and a standard Hill equation in the form of:

$$\frac{I}{I_{cont}}=\frac{A_{\max}}{1+10^{\left({LogIC}_{50}-x\right)n_{h}}}$$

where *I*_*cont*_ and *I* are the current magnitude in the absence and presence of a given concentration of drug (x; in Log(M)) respectively. $A_{\max}$ is the maximal inhibition, *IC*_*50*_ is the concentration of an inhibitor which inhibits 50% of the sensitive current, and $n_{h}$ is the Hill coefficient (using Prism 9.2.0 (GraphPad). Where data were best fit by the sum of two Hill equations, modified equations were totaled in which $A_{\max}$ was multiplied by the fraction of the inhibition ($A_{frac}$) which each component (a and b) accounted for:

$$\frac{I}{I_{cont}}=\frac{A_{\max}\times A_{frac,a}}{1+10^{\left({LogIC}_{50,a}-x\right)n_{h,a}}}+\frac{A_{\max}\times A_{frac,b}}{1+10^{\left({LogIC}_{50,b}-x\right)n_{h,b}}}$$

### Data analysis and statistics

All experimental values are described in text as mean ± standard error of mean (SEM). Bar charts present all individual data points and the standard deviation (SD) to convey variation as previously (56). The *t* tests and one-way ANOVAs were performed using Prism 9.2.0 (GraphPad) and Tukey’s post-hoc test was used to make multiple comparisons where appropriate. *p* values of less than 0.05 were taken as being statistically significant and are indicated by ∗. *p* values of less than 0.01, 0.001 and 0.0001 are signified by ∗∗, ∗∗∗, and ∗∗∗∗, respectively. This article contains supporting information.

## Data availability

Data are available upon request to the corresponding author.

## Supporting information

This article contains supporting information.

## Conflict of interest

The authors declare that they have no conflicts of interest with the contents of this article.
