# Supplementary material for: Preferential formation of human heteromeric SK2:SK3 channels limits homomeric SK channel assembly and function
Source: J Biol Chem. 2022 Dec 9;299(1):102783. doi: 10.1016/j.jbc.2022.102783 (PMC9841042; doi:10.1016/j.jbc.2022.102783)
Supplement: Supplementary information [file mmc1.docx]

**Supplementary information for**

**Preferential formation of human heteromeric SK2:SK3**

**channels limits homomeric SK channel assembly and function**

By

Andrew S. Butler^1^, Jules C. Hancox^1^*, Neil V. Marrion^1*^

^1^ School of Physiology, Pharmacology and Neuroscience, Medical Sciences Building, University Walk, Bristol, BS8 1TD. United Kingdom.

* Authors for correspondence

[n.v.marrion@bristol.ac.uk](mailto:n.v.marrion@bristol.ac.uk)

[Jules.Hancox@bristol.ac.uk](file:///C:\Users\andre\Downloads\Jules.Hancox@bristol.ac.uk)

Additional experiments were performed to ensure that the transfection approach used (500 ng WT-hSK2 + 100 ng WT-hSK3) was not responsible for the recorded preferential formation of heteromeric channels. 833 ng WT-hSK2 + 166 ng WT-hSK3 (*n* = 8) produced currents similar in size to those recorded when using 500 ng + 100 ng (*n* = 24), also matching those of homomeric hSK2-mediated currents (*n* = 21). These were significantly smaller than those produced by homomeric hSK3 channels (*n* = 18; Fig. S1*A*). The same results were seen using equal quantities of each subunit DNA (500 ng; *n* = 10; Fig. S1*A*). Additionally, reducing the quantity of WT-hSK3 DNA from 1 µg to 0.1 µg in the transfection mixture did not affect hSK3 current size (*n* = 11; Fig S1*B*). These data suggest that under all experimental conditions, maximal expression of SK channel subunits occurred and they indicate that the transfection ratio used is not responsible for the data presented.

|  |
| --- |

**Figure S1: Effects of transfection conditions on current size. A)** Current density in HEK293 cells following transfection with 1 µg WT-hSK2; 1 µg WT-hSK3; or co-transfection with WT-hSK2 and WT-hSK3 under different transfections conditions. Ratios represent the mass (in ng) of SK channel subunit DNA included in the transfection mixture. During the 500:100 transfection, 400 ng empty pcDNA vector was included to maintain a total of 1 µg DNA. **B)** Current density in HEK293 cells following transfection with 1 µg or 0.1 µg WT-hSK3. When 0.1 µg was used, 900 ng empty pcDNA vector was included to maintain a total of 1 µg DNA. Reducing the quantity of SK subunit DNA had no effect on current density. In all transfections, 200 ng eGFP was also used. In ‘A’ and ‘B’, currents were elicited using voltage ramps and measured at -20 mV. Bar charts show mean ± SD with individual data points shown as open circles.
